# Community engagement in patient-centered outcomes research: Benefits, barriers, and measurement

**DOI:** 10.1017/cts.2018.341

**Published:** 2019-05-14

**Authors:** Linda Sprague Martinez, Kelsi Carolan, Arden O’Donnell, Yareliz Diaz, Elmer R. Freeman

**Affiliations:** 1 Boston University School of Social Work, Boston, MA, USA; 2 Center for Community Health Education Research and Service, Inc., Boston, MA, USA

**Keywords:** Delphi method, stakeholder engagement, community partnerships, patient centered outcomes research, PCORI, implementation, policy and community engagement

## Abstract

**Introduction:**

This study employed the Delphi method, an exploratory method used for group consensus building, to determine the benefits and challenges associated with community engagement in patient-centered outcomes research.

**Methods:**

A series of email surveys were sent to the Patient-Centered Outcomes Research Institute (PCORI)-funded researchers (*n* = 103) in New England. Consensus was achieved through gathering themes and engaging participants in ranking their level of agreement over three rounds. In round one, participant responses were coded thematically and then tallied. In round two participants were asked to state their level of agreement with each of the themes using a Likert scale. Finally, in round three, the group was asked to rank the round two themes based on potential impact.

**Results:**

Results suggested the greatest benefit of community engagement is that it brings multiple perspectives to the table, with 92% ranking it as the first or second most important contribution. Time was ranked as the most significant barrier to engaging community. Strategies to overcome barriers to community engagement include engaging key stakeholders early in the research, being kind and respectful and spending time with stakeholders. The most significant finding was that no researchers reported having specific measures to evaluate community engagement.

**Conclusion:**

Community engagement can enhance both research relevance and methodology when researchers are engaged in meaningful collaborations. Advancing the science of community engagement will require the development of evaluation metrics to examine the multiple domains of partnership.

## Introduction

The Patient-Centered Outcomes Research Institute (PCORI) was created through the Patient Protection and Affordable Care Act with the intention of elevating patient voice in research [1]. The PCORI mantra, “research done differently” translates to “funded research that can help patients and those who care for them make better-informed decisions about the healthcare choices they face every day, guided by those who will use that information” in the provision of their care [2]. Engagement can help ensure research is culturally relevant and aligned with community priorities and can help to sustain engagement and interest among those most impacted [3]. In addition, it can promote resource sharing and co-learning between community partners and researchers, increasing their capacity [4]. Importantly, such approaches can facilitate the translation of research into policy and practice [5].

There are well-documented challenges associated with engaging communities (both residents and organizations) in research. Differences in priorities as well as a lack of transparency can lead to disagreements between researchers and community partners [6]. Communication barriers can emerge as the result of disciplinary jargon and exclusionary language on the part of the researchers [7]. Power dynamics between researchers and partners, in this case patients and providers, can complicate interactions and impede the development of trusting relationships [8]. Real barriers exist between researchers and community partners, particularly in communities of color, which have been historically exploited by academic researchers and the medical community. These barriers may accumulate to render relationship development time-intensive work [9, 10].

Similarly to our previous work exploring community engagement in the Clinical Translational Science Award (CTSA) program, to inform testimony to the Institute of Medicine [11], we employed the Delphi Method (Delphi) to determine the benefits and challenges associated with community engagement in patient-centered outcomes research (PCOR). In this instance we defined community as patients and those who work with them in the broader community. The Delphi method, specifically, was designed to be an exploratory consensus-building tool [12]. It is used by researchers and practitioners from diverse disciplinary backgrounds and is particularly useful in situations in which face-to-face meetings are not possible. Delphi was a tool for soliciting the opinions of PCORI-funded researchers across New England to garner a sense of the barriers and facilitators associated with engagement in the context of PCOR.

This paper describes the Delphi process employed. The overall results are then discussed in the context of the literature. The study confirms previous findings related to the barriers to and benefits associated with community engagement in health research more broadly. Perhaps the most salient finding is the lack of emphasis that is being placed on the measurement and evaluation of community engagement in PCOR research.

## Methods

The Delphi method is an iterative process, requiring participants to engage with questions asked of them over time, through the use of consecutive surveys [12]. One of the key elements of the Delphi process is that in the second and third rounds, participants have the benefit of (1) seeing the input from all the other participants in the process and (2) having the group inform their personal input [12]. Participants are able to reflect on the topic at hand and incorporate the group’s knowledge, which results in a consensus in which every voice is heard.

Utilizing a list of PCORI-funded researchers obtained from the PCORI website, we identified the contact information for each principal investigator on the list of PCORI awardees. An electronic survey explaining the goal and objective of the Delphi was then sent to awardees (*N* = 103). Survey questions used to evaluate community engagement in the CTSA program were adapted by our PCORI Community Advisory Board. A total of three rounds were conducted by email survey, each time allowing participants 14 days to respond. All rounds were sent to the original recipients regardless of whether they responded in previous rounds. In each round, the respondent was asked to identify their state, gender, educational background, and role on the PCORI project: (1) Principal Investigator, (2) Project Lead, (3) Project Coordinator, (4) Research Assistant, (5) Community Stakeholder, or (6) a specific other.

The first round of the survey consisted of six open-ended questions exploring participant perceptions of community engagement in PCOR. More specifically, the questions examined: the role of community stakeholders in PCOR; how institutional and community politics impact community engagement; barriers to community engagement; strategies for overcoming engagement barriers; and methods for evaluating community engagement in PCOR. Reports were generated using Qualtrics. Open-ended responses were reviewed by two researchers, who thematically coded participant responses by question. Researchers then met to review and reconcile the themes, assuring the reliability of results. Round one themes were listed as responses for each of the six original items. Participants were asked to rate their level of agreement on a Likert scale ranging from disagree strongly to agree strongly. The third and final round included all of the responses from round two, and asked participants to rank the responses in order of importance.

## Results

### Round One

A total of 47 individuals participated in the first round of the Delphi, with the majority of participants representing projects based in Massachusetts (*n* = 36). The remaining participants were from Connecticut (7), Vermont (2), New Hampshire (1), and Maine (1). We anticipated this would be the case, given that 76.7% of the sample was based in Massachusetts. All but one participant in the first round identified as a principal investigator or project lead. The remaining individual identified as a research assistant. The gender split was fairly even, with 24 females and 21 males.

Participants were asked to describe the benefits of engaging community. It was reported that engagement increases researcher accountability to the community. Moreover, community engagement was seen as a process that ultimately enhances the research by bringing multiple perspectives to the table and improving the research’s relevance, as well as credibility, by ensuring alignment between researcher and community goals. There was agreement across participant responses that community engagement may increase both researcher and community capacity. Finally, participants reported engagement reduces inequitable power dynamics between researchers and stakeholders. A sample of illustrative quotes can be seen in Table 1.

**Table 1.** The benefits of stakeholder engagement

Illustrative quotes
*By engaging community stakeholders, organizing periodic meetings and cross-training activities both the community partners and the research team are able to learn from each other and develop a perspective that is based on the lived experience of individuals in the community.*
*Fosters new relationships and collaborations that build new opportunities among all stakeholders.*
*Research is undertaken on topics that matter to people with MS [multiple sclerosis]. Research study designs may be improved so that the results become more relevant to people with MS. Research studies are enabled by the enthusiastic participation of community members. People with MS understand the value of the community and are more likely to participate – making more people-powered research possible.*
*Each stakeholder holds the key to a certain part of a puzzle. Collectively engaging with a broad spectrum of stakeholders increases the probability that we can solve the problem.*
*“It’s not about us without us!” If we want to know what matters; what is working; what could be working; what is not working, we need to listen to the people who experience the impact of our choices.*
*We are able to break down power dynamics and focus on issues important to the community and work toward goals important to the community.*

Participants were asked to share the ways in which politics – institutional, community, or otherwise – impact community engagement. This question proved problematic. Some reported it was unclear, while others described it as a “loaded” question. Those who responded implicated institutional racism as a central way in which politics impacts community engagement. Additional themes that emerged in response to this question included: conflicting interests, bureaucracy, time, funding, lack of trust, and community politics. The stigma associated with particular health areas (such as mental health and substance use disorders) impacts community engagement. Examples of themes can be seen in Table 2.

**Table 2.** Politics that impact engagement

Themes	Illustrative quotes
Conflicting interests	*Some individuals have different priorities of what they think is important and steer the group in self-serving directions.*
Bureaucracy	*There are some policies about how stakeholders are compensated that can affect sustainability of engagement.* *In order to participate in our study, we are required to file paper work for each of the community partners. The completion of these documents can be a challenge when new community partners join and when the study is moving forward.* *Hiring challenges if someone has an incarceration history; pay scale; having voice and recognition in some settings.*
Time	*When engaging community-based organizations the biggest barrier is the time commitment. Small non-profits have limited staff and tight budgets that leave people wearing multiple hats. Volunteering time to participate in meetings and other additional work can be a hard sell, even when the organization or individuals have a strong interest.* *…Budgets in both community health centers and in clinics are bare-bones so they have very limited infrastructure or time for capacity development…*
Lack of trust	*…There is a huge power differential between the individual and the institution, impacts the willingness of people to trust the process.* *Structural racism and our challenged history with American Indian communities in the USA has led to poorer health outcomes among American Indian populations and we now have to work to re-build trust and support for carrying out meaningful and impactful research that will benefit the communities in which we work.*
Community politics	*…Internal community-specific politics may get in the way.* *There are always “turf” issues to consider when bringing groups together…* *Politics has a huge impact. If there are people/ organizations that don’t like each other or have conflicts of interest…*
Stigma	*Our topic is related to mental health, which is itself a political as well as generally stigmatized topic. Thus, gaining buy-in can be challenging.*

Additional barriers to community engagement described by participants included: time, staffing limitations, funding, bureaucracy, the challenges of educating partners, and insufficient engagement expectations or superficial engagement, in which partners are tokenized. In addition, the prevalence of jargon and technical language in research was seen as creating communication barriers between researchers and community partners. Limited English proficiency was also seen as a barrier, along with deep-seated community mistrust and fear of exploitation. Finally, participants described the difficulty of identifying and engaging patients of color as a barrier.

Recommendations for addressing the barriers to community engagement included increasing communication, spending time getting to know community partners, and developing structures for shared decision-making and shared goal setting. Participants recommended engaging community early and across all phases of the research, taking steps to ensure equitable funding and compensation, and increasing investigator education related to best practices for collaborating with communities. Participants specifically indicated transparency and flexibility on the part of the researcher, as well as being kind, genuine, and respectful to partners were strategies for overcoming engagement barriers. Finally, participants were invited to describe how they are evaluating community engagement, including the use of metrics. No metrics were identified, although some participants reported working on measure development. Evaluative methods reported include: qualitative interviews, process measures and anonymous surveys, as well as tracking participation.

### Round Two

Round two had a total of 34 responses. As was the case with round one, the majority of respondents (*n* = 24) were from Massachusetts. Twenty-nine participants were principal investigators or project leads and two were project coordinators; those remaining were community partners (*n* = 3). In round two, there were slightly more women than men (*n* = 20 and *n* = 14, respectively). The majority of the participants agreed or strongly agreed with the themes identified regarding benefits of engaging community stakeholders. The greatest agreement was with the statement: community engagement brings multiple perspectives to the table, which enhances the research. The vast majority of participants were in agreement that engagement increases researcher accountability to community, improves the appropriateness of the research and lends credibility to the research. Finally, a majority agreed community engagement increases the capacity of the research team. The least amount of strong agreement was with the statement community engagement reduces inequitable power dynamics. A summary of responses can be found in Table 3.

**Table 3.** The benefits of engagement

To what extent do you agree that each of the following is a benefit associated with community engagement?					
	Strongly disagreed % (*n*)	Somewhat disagree % (*n*)	Neutral % (*n*)	Somewhat agree % (*n*)	Strongly agree % (*n*)
Increases researcher accountability to the community	6.06% (2)	6.06% (2)	6.06% (2)	36.36% (12)	45.45% (15)
Brings multiple perspectives to the table which enhances the research	6.06% (2)	0.00% (0)	0.00% (0)	24.24% (8)	69.70% (23)
Improves the appropriateness of the research	6.06% (2)	0.00% (0)	18.18% (6)	33.33% (11)	42.42% (14)
Lends credibility to the research	6.06% (2)	3.03% (1)	21.21% (7)	36.36% (12)	33.33% (11)
Increases the capacity of the research team and the community	6.06% (2)	3.03% (1)	24.24% (8)	27.27% (9)	39.39% (13)
Reduces inequitable power dynamics	6.06% (2)	6.06% (2)	24.24% (8)	45.45% (15)	18.18% (6)

As indicated in Table 4, agreement with the factors impacting stakeholder engagement identified in round one was not unanimous, with the exceptions of time, bureaucracy, and the distribution of funding and compensation. Responses were mixed regarding the extent to which participants agreed on the impact of institutional racism, institutional hierarchy, conflicting interests, and stigma. Most participants disagreed that resistance to outcomes research impacts stakeholder engagement. All participants agreed that time is a barrier to engagement, as are staffing limitations, funding, engaging patients of color, and inconsistent engagement expectations or superficial engagement. Most participants agreed that communication barriers existed due to jargon and technical language. A smaller majority of participants agreed limited English proficiency is a barrier, as well as stakeholder mistrust and educating stakeholders.

**Table 4.** Factors impacting engagement

To what extent do you agree that each of the following factors impacts stakeholder engagement?					
	Strongly agree % (*n*)	Somewhat agree % (*n*)	Neutral % (*n*)	Somewhat disagree % (*n*)	Strongly disagreed % (*n*)
Institutional racism	12.50% (4)	34.38% (11)	21.88% (7)	21.88% (7)	9.38% (3)
Conflicting interests	9.38% (3)	50.00% (16)	12.50% (4)	21.88% (7)	6.25% (2)
The distribution of funding and compensation	21.88% (7)	50.00% (16)	12.50% (4)	12.50% (4)	3.13% (1)
Bureaucracy	12.50% (4)	62.50% (20)	12.50% (4)	6.25% (2)	6.25% (2)
Time	56.25% (18)	31.25% (10)	9.38% (3)	0.00% (0)	3.13% (1)
Resistance to outcomes research	0.00% (0)	18.75% (6)	21.88% (7)	43.75% (14)	15.63% (5)
Institutional hierarchy	6.25% (2)	37.50% (12)	31.25% (10)	21.88% (7)	3.13% (1)
Stigma associated with particular health areas (*i.e.,* mental health and substance use disorder)	6.25% (2)	34.38% (11)	31.25% (10)	21.88% (7)	6.25% (2)

Overall, participants agreed with the strategies identified in round one for overcoming engagement barriers. The strongest level of agreement was indicated for the following strategies: engage stakeholders early and across all phases of the research (75% strongly agreed and 18.75% somewhat agreed); be kind, genuine, and respectful (75% strongly agreed and 18.75% somewhat agreed); and spend time getting to know community stakeholders (71.88% strongly agreed and 21.88% somewhat agree). Participant responses also indicated fairly high agreement that developing structures for shared decision-making and shared goal setting, as well as assuring equitable funding and compensation are effective strategies for overcoming engagement barriers.

Finally, participants agreed with the identified strategies for evaluating community engagement, with the majority indicating that they strongly or somewhat agree that qualitative interviews (96.88% agreement), anonymous surveys (90.63), and tracking participation (81.26%) are effective measures. Process measures yielded the lowest level of agreement, with more than three quarters of participants strongly or somewhat agreeing that process measures are an effective strategy for evaluating community engagement.

### Round Three

There were 36 participants in round three. As was the case with rounds one and two, the majority of respondents were in Massachusetts (25). Thirty of the 36 participants identified as a principal investigator or project lead; the remaining participants identified as community engagement, patient co-investigators, and patient research partners. More women than men responded (23 and 11, respectively). One participant identified as other and one did not complete the gender item. Participants were asked to rank items identified in rounds one and two in terms of their relative importance. Participants indicated bringing multiple perspectives to the table which enhances research is the greatest potential benefit associated with community engagement, with 65.52% of participants ranking this item first and 27.59% of participants ranking it second. The largest proportion of participants ranked improves the appropriateness of the research as offering the second most potential benefit, although agreement was lower, with 20.69% ranking this item first, and 31.03% ranking it second. The statement that engaging community stakeholders as a potential benefit by lending credibility to the research was ranked the lowest.

Participants were asked to rank items identified in rounds one and two as impacting community engagement in order of most to least impactful. Time was considered to have the greatest impact, with 48.28% of respondents ranking it first. Bureaucracy was also considered highly impactful, with 13.79% of participants ranking it first and 27.59% ranking it second. Stigma and institutional racism were ranked as least impactful. Participants were also asked to rank which items identified in the first rounds presented the greatest barrier to community engagement. Time was again considered the greatest barrier to engagement, with the largest portion of participants ranking it first compared to other items (42.86). Nearly 18% (17.86) of participants ranked funding as the greatest barrier, and 17.86% of participants designated staffing limitations as the second greatest barrier (the highest level of agreement regarding the second greatest barrier after time, which was ranked by 28.57% as the second greatest barrier). Results indicated less concern about barriers associated with limited English proficiency, mistrust, and fear of exploitation, with these items consistently ranking low across participants.

With respect to strategies for overcoming engagement, there was not a great degree of consensus among the group as to which strategies might be most effective. Twenty-five percent of participants ranked increase communications with community first, 21.43% ranked spending time getting to know community stakeholders first, and 25% ranked being kind, genuine, and respectful as first, the most effective strategy for overcoming engagement barriers. Interestingly, although there was agreement in round two about the importance of being kind, genuine, and respectful – and 25% ranked this item first – a quarter of respondents also considered it to be the least effective strategy for overcoming engagement barriers. The majority of participants (71.43%) felt that increasing investigator education would be the least effective strategy.

Finally, participants ranked qualitative interviews as the most effective strategy for evaluating community engagement among the response options, with 68% of participants ranking this item first. Process measures, tracking participation, and anonymous surveys were ranked lower, with 40% of participants ranking tracking participation second, 48% ranking process measures third, and 44% ranking anonymous surveys as the least effective strategy.

## Discussion

Participants reached a high level of consensus regarding the greatest potential benefit offered by community engagement in research: that doing so brings multiple perspectives to the table, which ultimately enhances the research. This finding aligns with the extant literature examining researchers’ perspectives on the benefits of PCOR. Researchers interested in engaging patients in the research process may be most motivated by the desire to gain improved understanding of patients’ unique perspectives on and experience with a disease [13]. Selby *et al*. [14] discuss findings from surveys of PCORI-funded researchers indicating community engagement has enhanced the research process in tangible ways. More specifically, engagement has aided in identifying research questions, appropriate study designs, interventions and outcomes most relevant to patients, in addition to enhancing both recruitment and retention.

Participants also agreed that community engagement benefits the research process by improving the overall relevance of the research. These findings echo existing literature indicating community engagement in the context of PCOR is widely considered to improve the relevance of research [15–17]. Engagement orients the research toward the questions and concerns most valuable to patients and other stakeholders [14, 16]. Thus, community engagement may result in research that is more meaningful, from the questions selected to the outcomes examined [13, 14, 16].

Consistent with the extant literature, time was identified as the most significant barrier to community engagement. In a survey of PCORI-funded researchers, Forsythe *et al.* [13] found researchers considered lack of time – on the part of both researchers and community partners – one of the most significant barriers to community engagement in research. Selby *et al*. [14] report researchers applying for PCORI funding have indicated that establishing relationships with community partners, including patients, within the time restrictions of submitting a grant proposal can present a challenge. In addition to time, Forsythe *et al.* [13] found staffing limitations in the form of limited research team resources and training to be significant barriers to community engagement.

Participants in the present study pointed to bureaucracy as impacting community engagement, in addition to funding and staffing limitations. Carman and Workman [15] discuss how the long-standing privileging of health professionals’ perspectives has led to institutional environments with multiple systemic barriers to partnering with community partners. Considerable restructuring and challenging of existing power dynamics has had to occur in order to open pathways for community engagement, and additional infrastructure is needed.

Although findings indicated less agreement with regard to how to best overcome these barriers to stakeholder engagement, the results did point toward increased communication with community stakeholders and spending time getting to know stakeholders as potentially important strategies. Communication strategies are generally considered central to addressing the challenges of engaging community in the research process [13, 15]. Carman and Workman [15] describe clear communication as key to adequately preparing partners to engage in the research process. Researchers leading PCORI-funded projects have highlighted specific communication strategies as essential to overcoming certain barriers and improving the engagement process, including ensuring partners have the opportunity to ask questions, making an effort to stay consistently in contact with them, meeting in person, avoiding technical or medical jargon, and ensuring that opportunities for socializing are allowed to take place [13]. This last strategy closely reflects this study’s finding that spending time getting to know community partners is important.

Perhaps the most salient finding was that among PCOR investigators and partners we engaged in the Delphi, none reported having specific measures to evaluate community engagement. Although, some reported working on developing metrics, most described using qualitative interviews, process measures, tracking participation, and anonymous surveys. One challenge for PCORI researchers will be to avoid what Goodman and Thompson [18] term “symbolic participation,” where partners are allowed to hear plans and have a voice, but those voices do not carry the weight of influence. Advancing the science of community engagement in PCOR will require systematic evaluation and the development of metrics which assess a variety of domains such as levels of collaboration, co-learning, trust between community partners and researchers, and shared decision-making authority. Although successful engagement in a single project is admirable, PCORI researchers will benefit from drawing on the lessons and best practices of community-based participatory researchers as they work to develop new metrics.

Although institutional racism, hierarchy, and stigma came up as barriers in round one, they were not prioritized in later rounds. This finding was inconsistent with the literature which points to systems of oppression, particularly racism, as impacting community engagement. It may be that those who raised the concern in the first round did not participate in subsequent rounds, which is a limitation of the Delphi method. It may have also been the makeup of our sample. Although we encouraged participants to share the survey link with their partners, community participation was minimal at best. As such, the results presented represent the views of principal investigators and project leads for the most part, who are researchers in academic medical settings. The views of community partners are likely to be different from those of researchers. An additional limitation to this work was the fairly response rates low in each round and not all participants completed all of the items. We have included the number of responses in addition to the percent of respondents in the tables to provide a more accurate picture for the reader. Despite limitations, these data provide an important starting point for understanding the perceived benefits and barriers to community engagement in PCOR. More importantly it highlights the need for metrics to better understand community engagement in PCOR.

## Conclusions

The challenges to community engagement identified by PCORI-funded researchers in our sample are consistent with the literature on engagement in PCOR. Findings are also well aligned with the challenges experienced by community engagement researchers in the CTSA program. Community engagement can enhance the both the research relevance and the methodology. This can only happen when researchers are engaged in meaningful collaborations with stakeholders which calls for intentionality and takes time. Advancing the science of community engagement in PCOR will require systematic evaluation and the development of metrics to examine the multiple domains of partnership. There is a need for increased research infrastructure in the community; in addition, academic research institutions will need to restructure to facilitate genuine engagement and participation.
